# Prognosis of ulcerative colitis colorectal cancer vs. sporadic colorectal cancer: propensity score matching analysis

**DOI:** 10.1186/s12893-017-0224-z

**Published:** 2017-03-21

**Authors:** Yoon Dae Han, Mahdi Hussain Al Bandar, Audrius Dulskas, Min Soo Cho, Hyuk Hur, Byung Soh Min, Kang Young Lee, Nam Kyu Kim

**Affiliations:** 1 Division of Colorectal Surgery, Department of Surgery, Yonsei University College of Medicine, 50 Yonsei-ro Seodaemun-gu, Seoul, 120-752 Korea; 2 Department of Oncosurgery, National Cancer Institute, Vilnius, Lithuania

**Keywords:** Surgery, Ulcerative colitis, Colorectal cancer, Sporadic cancer

## Abstract

**Background:**

Ulcerative colitis (UC) harbours a high risk of UC-associated colorectal cancer (UCCC), which is important cause of morbidity and mortality in patients with inflammatory bowel disease. Overall Survival (OS) of patients with UCCC has not been addressed well in the literature. Thus, we compared oncologic outcome of UCCC and sporadic colorectal cancer (SCC) using propensity score matching analysis.

**Methods:**

Propensity score matching was performed for 36 patients, a 1:1 matching method stratified into 18 in UCCC and 18 patients in SCC. Matched variables were sex, age, body mass index, tumour stage, histology, preoperative carcinoembryonic antigen (CEA) level, and adjuvant treatment status. Patients with SCC or UCCC were retrospectively retrieved from our database from March 2000 to December 2015. All patients had undergone either oncological segmental resection or total proctocolectomy.

**Results:**

The majority of cancers were found in the sigmoid colon. Total proctocolectomy was performed only in the UCCC group; however, half of the UCCC group underwent a standard operation. Five cases of postoperative complication occurred within six months in the UCCC group compared to one case in the SCC group. There was no significant difference in recurrence rate (*p* = 0.361) or OS (*p* = 0.896) between the arms.

**Conclusion:**

UCCC showed more postoperative complications than SCC, and equivalent oncology outcome, however the difference was not statistically significant. This study represents an experience of a single institution, thus further randomized studies are required to confirm our.

## Background

Ulcerative colitis (UC) is a chronic inflammatory disease of the large bowel for which the aetiology is not fully understood [1]. This disease harbours a high risk of associated colorectal cancer (UCCC). This is the most important cause of morbidity and mortality in patients with inflammatory bowel disease (IBD) [2], accounting for 10-15% of deaths in IBD patients [3]. However, the real impact of colorectal cancer (CC) is still debated in the literature. Fifteen years ago, Eaden et al. published a meta-analysis reporting that the cumulative risk of CC in patients with UC was 2% at 10 years [4]. A later meta-analysis by Lutgens et al. reviewed eight studies published from 1988 to 2009 and showed that the risk of CC was increased in IBD but was not as high as reported in earlier studies; the pooled standardised incidence rate in their study was 1.7 [5]. Recently, Castano-Milla and colleagues published their systemic review and meta-analysis [6], in which they concluded that the risk of developing CC among patients with UC has decreased steadily over the last six decades and was 1.58 per 1,000 patient-years. Another recent meta-analysis showed a 2.4-fold higher rate of CC in UC patients compared to the overall population [7].

Previous studies have reported several clinical and pathogenetic differences between sporadic CC (SCC) and UCCC. It is known that CC occurs about 30 years earlier than UCCC in UC patients [4]. UC patients are known to have higher rates of multifocal, right-sided tumours and mucinous or signet ring cell carcinomas, which are associated with poor prognosis [8, 9].

The survival of patients with UCCC was not discussed in previous reports. Early studies showed five -year survival rates up to 30% [10–13]; however, the overall survival (OS) for patients with SCC at the same period was similar. Recent studies have shown much higher survival rates and no significant difference between UCCC and SCC patients [14]. In contrast, some other studies have reported poorer survival for UCCC patients than for patients with SCC [8, 15–18]. The authors explained the poorer survival in UCCC patients by an unfavourable stage distribution, since a greater proportion of UCCC patients had distant metastasis at the time of cancer diagnosis. The existing data are conflicting and heterogeneous, as patient collectives vary regarding factors such as follow-up period, matching criteria, and stage distribution.

Because of the relatively low prevalence, little is known about the differences in prognosis between SCC and UCCC. In this study, we compared two cohorts of UCCC and SCC patients in terms of long-term outcome and recurrence rate using propensity score matching analysis.

## Methods

### Patients

Data for all patients who underwent surgical treatment for UCCC and SCC in the Department of Surgery at Yonsei University between March 2000 and December 2015 were collected retrospectively. All patients diagnosed with colon cancer due to sporadic or ulcerative colitis have been enrolled in our analysis. However, to achieve an accurate analysis we carried out propensity cross match analysis. A total of 36 patients divided according to aetiology of colon cancer into 18 patients by UCCC and 18 patients were SCC. All patients have been matched following these criteria: age at surgery (±5 years), sex, body mass index (BMI), tumour stage (according to American joint commission for cancer), histology, preoperative CEA level, and adjuvant treatment. Patient demographics, co-morbidities, detailed operative information, histopathologic tumour features, and follow-up data were obtained by medical record review. The primary outcome for comparison was OS. Recurrent disease was defined if a suspected lesion became apparent by imaging modalities after a period of 6 months of undetectable disease, including local recurrence in the same location as the primary tumour, regional recurrence, and distant recurrence in other organs such as liver and lungs. Board-certified colon and rectal surgeons at a tertiary referral centre participated in performing designed surgery. Resections for CC included segmental proctectomy or extended resection by total proctocolectomy.

### Statistical analysis

Categorical variables are expressed as percentage and compared by Fisher’s exact test or Chi-square test as appropriate. Continuous variables are presented as mean ± standard error of the mean or median and interquartile range (IQR) per group and are compared using two-tailed Student’s *t*-test. Survival curves for 1, 3, 5, and 10 years were generated using the Kaplan-Meier method, and survival was compared by log rank and Breslow tests. Survival percentages per measured time point are reported with 95% confidence interval (CI). Significance was set at *p* < 0.05. Calculations were performed using SPSS Statistics for Windows.

## Results

**Fig. 1:**
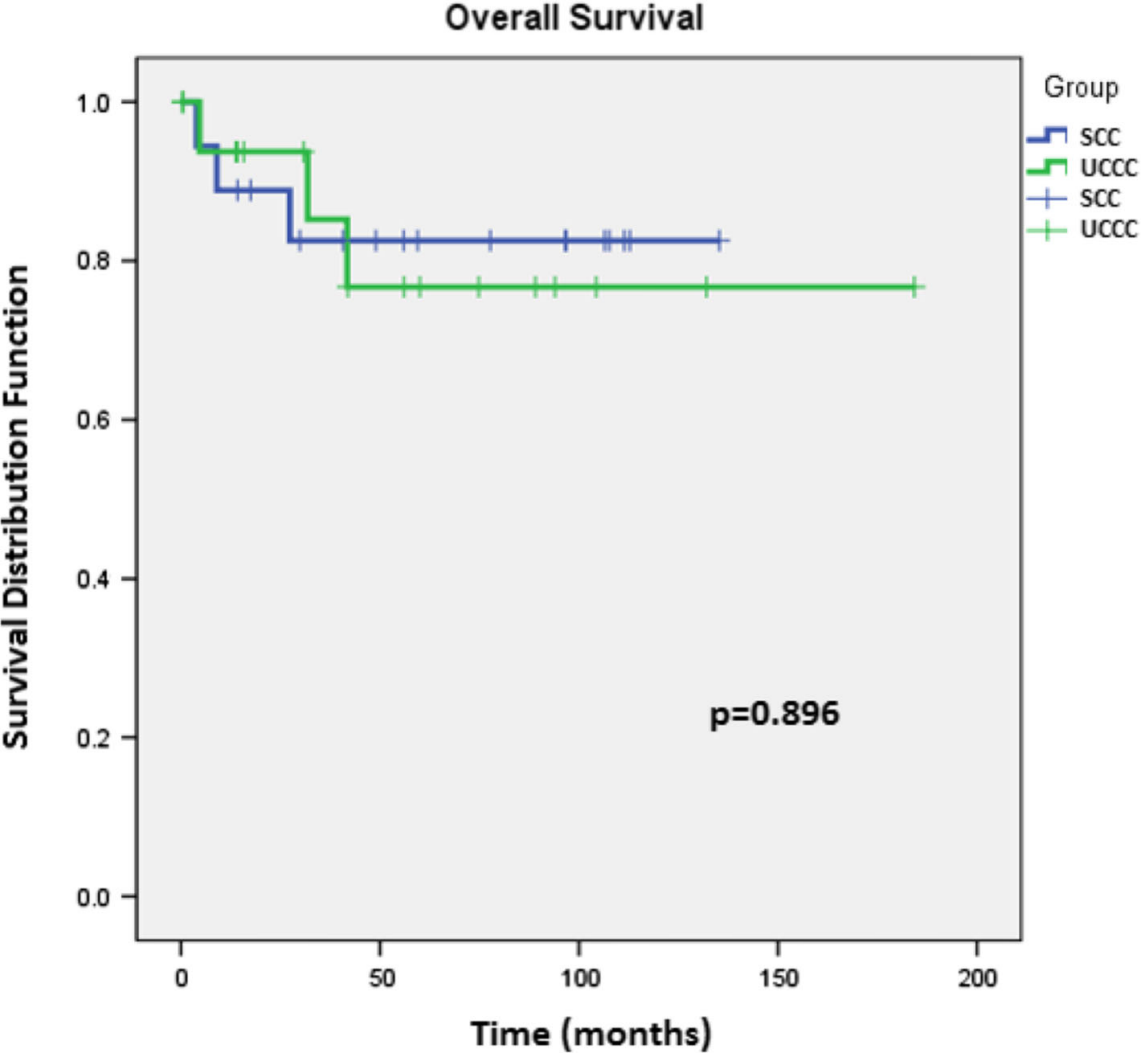
Kaplan–Meier estimates of overall survival and 5- and 10-year overall survival rates of 18 patients with ulcerative colitis-associated carcinoma and 18 patients with sporadic colorectal carcinoma. SCC – sporadic colorectal cancer; UCCC – ulcerative colitis-related colorectal cancer SCC 114.05 ± 11.13, 95% CI (92.23 – 135.87) UCCC 147.89 ± 18.55, 95% CI (111.53 – 184.24) Log rank: *p* = 0.896 Breslow: *p* = 0.932

Out of 36 patients, 18 (50%) had SCC and 18 (50%) had UCCC. Patient characteristics of BMI, age, sex, pathological stage, preoperative CEA level, and tumour location were matched in both groups (Table 1). Adjuvant chemotherapy was administered equally to both groups. The majority of cancers were found in the colon: 15 (83.3%) for each of SCC and UCCC. Most patients in the SCC group presented with stage II cancer (9 patients, 50%), whereas in the UCCC group, 6 patients (33.3%) had stage II and 7 (38.9%) had stage III disease. Total proctocolectomy was performed significantly more often in the UCCC group (9 vs. 0 patients, *p* = 0.001). UCCC showed more postoperative complications than SCC (5 vs. 1), although this was not significant (*p* = 0.634). A relatively higher local recurrence rate was observed in SCC (5.6% [1 patient] vs. 0 in the UCCC group), but the overall recurrence was not significantly differ (*p* = 0.361) (Table 2). There was no significant difference in five-year OS between the two arms (Fig. 1).

**Table 1:** Characteristics and perioperative findings of patients diagnosed with colorectal cancer

Variables	Sporadic (*n* = 18)	UC (*n* = 18)	*P*-value
Age at surgery (years)	52 (30–73)	51 (31–68)	0.866
Sex			0.735
Male	7 (38.9%)	8 (44.4%)	
Female	11 (61.1%)	10 (55.6%)	
BMI (kg/m^2^)	23.23 ± 3.36	22.38 ± 1.47	0.328
Stage			0.593
1	4 (22.2%)	5 (27.8%)	
2	9 (50%)	6 (33.3%)	
3	5 (27.8%)	7 (38.9%)	
Adjuvant chemotherapy			1.0
Yes	12 (66.7%)	12 (66.7%)	
No	6 (33.3%)	6 (33.3%)	
Histology			0.388
WD	3 (16.7%)	5 (27.8%)	
MD	13 (72.2%)	9 (50%)	
PD	2 (11.1%)	4 (22.2%)	
Carcinoembryonic antigen (CEA)	2.27 ± 2.28	2.55 ± 1.79	0.684
Location			1.0
Rectum	3 (16.7%)	3 (16.7%)	
Colon	15 (83.3%)	15 (83.3%)	

All values are represented as *n* (%) or mean ± SD

*UC* ulcerative colitis, *WD* well differentiated, *MD* moderately differentiated, *PD* poorly differentiated

**Table 2:** Operative variables and short- and long-term results of patients undergoing colonic or rectal resection

Variables	Sporadic CC (*n* = 18)	UCCC (*n* = 18)	*P* value
Operation method			0.735
Open	8 (44.4%)	7 (38.9%)	
MIS	10 (55.6%)	11 (61.1%)	
Operation name			0.001
Total proctocolectomy with end ileostomy	0 (0%)	1 (5.6%)	
Total proctocolectomy with J-pouch anal anastomosis	0 (0%)	4 (22.2%)	
Total proctocolectomy with ileo-anal anastomosis	0 (0%)	4 (22.2%)	
Others	18 (100%)	9 (50%)	
Complication			0.624
Obstruction	1 (5.6%)	4 (22.2%)	
Rectovaginal fistula	0 (0%)	1 (5.6%)	
Recurrence			0.361
Local	1 (5.6%)	0 (0%)	
Systemic	2 (11.1%)	2 (11.1%)	

All values are represented as *n* (%)

*MIS* minimal invasive surgery, *UC* ulcerative colitis, *UCCC* ulcerative colitis-related colorectal cancer

## Discussion

In this study, we compared the prognosis of patients with UCCC or SCC in a matched pair analysis that including one of the largest cohorts of UCCC patients to date [14]. Prognosis was similar in the two groups, with a trend toward higher recurrence rate in the SCC group.

UCCC patients are typically younger and more frequently have multiple cancerous lesions and a macroscopically permeating pattern of spread, including mucinous or signet ring cell carcinomas, compared with SCC. The advanced stage at presentation results in a less favourable outcome for UCCC patients [2, 7, 8].

Chronic inflammation leads to the activation of sequins, which in turn leads to CC in UC patients. Colonic mucosa in UC exhibits high levels of chromosomal abnormalities clustered in regions of histological abnormality, and genomic derangements are a prominent feature of preinvasive neoplasia [1]. The same genetic and signalling pathways involving Wnt, β-catenin, K-ras, p53, and transforming growth factor (TGF)-β can be found in SCC and UCCC; however, a difference in activation timing between SCC and UCCC has been reported [3]. Mutations in p53 occur early in the adenoma-carcinoma sequence and are often detected in non-dysplastic or indefinite dysplasia in UC, whereas p53 mutations occur in the late phase in sporadic adenoma [19]. Microsatellite instability (MSI) is also relatively frequent in non-dysplastic inflamed epithelia, and transforming growth factor β receptor type II (TGFβRII) is one of the genes targeted by the MSI process in UCCC. Hypermethylation of *hMLH1*, *p16INK4a*, and *p14ARF* seems to precede dysplasia and contribute to the genetic alterations in UCCC [20]. Kinugasa et al. also showed that expression of claudin-1 was increased in UCCC and dysplasia compared with normal mucosa and is likely to be involved in neoplastic progression in UC patients [21].

Some previous studies have shown worse oncological outcomes in the UCCC patient group [8, 18, 22]. In a Japanese nationwide study, Watanabe et al. assessed 121 patients who had undergone surgery for UCCC. They found that, compared with patients with SCC, those with UCCC were younger and had a higher proportion of multiple cancer lesions, higher proportions of morphologically superficial type lesions and invasive type lesions, and higher proportions of mucinous or signet ring cell carcinomas. For stage III disease, patients with UCCC had a poorer survival rate than those with SCC (43.3% versus 57.4%, *P* = 0.0320); however, the OS was similar in earlier stages [8]. The UCCC-specific factors and different clinicopathologic patterns mentioned above are thought to result in an unfavourable prognosis [8, 23–25]. In a Danish cohort study by Jensen et al. 279 UCCC patients were compared to 71,259 SCC patients. The OS was significantly worse in the UCCC group. The negative effect of UC on the prognosis of CC was more pronounced in the first year after cancer diagnosis, in patients diagnosed with CC before the age of 70 years, in patients with UC duration of eight years or more, and in cancers with advanced or unknown stage [18]. In a recent matched pair analysis, 33 patients with inflammatory bowel disease-associated CC had a significantly reduced and markedly lower OS rate, as well as a notably increased recurrence rate, compared to sporadic CRC patients (48.6 vs. 67.1%, respectively) [22]. A single-centre retrospective study performed in 2016 compared OS and surgical technique in 27 patients with UC and rectal cancer and 54 patents with sporadic rectal cancer [14]. UCCC patients undergoing proctectomy demonstrated reduced disease-free survival (DFS) compared with those undergoing proctocolectomy and patients with sporadic rectal cancer undergoing proctectomy. The authors concluded that proctocolectomy should be considered the preferred surgical approach for colitis-associated rectal cancer.

Our study and some other retrospective single-centre studies showed no difference in OS [17, 26–28]. Our study was in agreement with the survival results of a Finnish study from 1998 that compared 33 UCCC patients to 122 patients with non-UCCC [17]. In two smaller retrospective case-matched cohort studies performed in a single tertiary centre by Kiran et al. [26] and Delaunoit et al. [27], the authors found no difference in OS between patients with IBD and CC with those with SCC. In a later study by Delaunoit, higher tumour grades and mucinous differentiation were more frequent among patients with UCCC. Kiran et al. [26] found no difference between two groups of UCCC and SCC patients in terms of local recurrence, DFS, or OS. In a recent large study from Germany, the authors matched UCC and SCC patients 1:1 [28]. Although they found more of the above mentioned risk factors in the UCCC group, the oncological outcome was the same in both groups. In a subgroup analysis of UCCC patients, female sex was associated with a significantly better prognosis. The authors assumed that oestrogens play a protective role in UCCC carcinogenesis.

Interestingly, some population-based studies have shown decreased mortality in patients with UCCC compared to those with UC. The authors explain this finding by long-term usage of 5-ASA [29]. The same study showed an increased risk of postoperative complications (postoperative deaths, septicaemia, other abdominal infections) in patients with UCCC. However, our study did not show any significant differences in the rate of complications.

Although total proctocolectomy with ileal pouch-anal anastomosis is the gold standard for UCCC, the pros and cons of rectal mucosectomy are still debated [30]. Whether this represents the optimal procedure for UC patients with SCC remains questionable, as the functional quality of life differs substantially between patients with proctocolectomy and those with partial resection. In elderly patients with poor anal function, surgical procedures should obviously be selected based on overall considerations including prognosis of the cancer, degree of inflammation with colitis, and potential requirements for future treatment. Uchino et al. have reported two cases with mild inflammation that underwent segmental colon resection [31]. Some case reports even show the usefulness of prognostic factors for the possibility of segmental resection [5].

Recent studies have shown the importance of surveillance programs in patients with long lasting UC [32, 33]. The authors concluded that colonoscopy results in diagnosis of cancer at earlier stages and younger ages. In addition, premalignant conditions can also be identified [32]. Most international guidelines recommend surveillance for UC using colonoscopy every 1-2 years over 8-10 years after the onset of UC diagnosis [34–39].

Our study has some limitations. First, the single centre, relatively small size, and retrospective nature of the study might weaken the significance of our findings. Second, there may be selection bias because not all patients who were diagnosed with UC and CC in the study period were included in the study, although we performed matched analysis for patient sex, age, body mass index, tumour stage, histology, preoperative CEA level, and adjuvant treatment status.

## Conclusion

In conclusion, our data suggest that patients with UCCC have the same prognosis as patients with SCC. All other factors related to UC (e.g., UC treatment before and after CC, disease activity and duration) and CC (e.g., aggressive tumours, surgical techniques, and surgical complications), as well as a number of other factors should be investigated in order to rule out confounding factor that might be of prognostic value (e.g., co-morbidity, co-medication, hereditary factors). Therein, well clinical trial is required to address this on going debates in the future.

## Abbreviations

BMI: Body mass index
CC: Colorectal cancer
CEA: Carcinoembryonic antigen
IBD: Inflammatory bowel disease
IQR: Interquartile range
OS: Overall Survival
SCC: Sporadic colorectal cancer
UC: Ulcerative colitis
UCCC: Ulcerative colitis -associated colorectal cancer

## References

[1] Kulaylat MN, Dayton MT (2010). Ulcerative colitis and cancer. J Surg Oncol.

[2] Ullman TA, Itzkowitz SH (2011). Intestinal inflammation and cancer. Gastroenterology.

[3] Lakatos PL, Lakatos L (2008). Risk for colorectal cancer in ulcerative colitis: changes, causes and management strategies. World J Gastroenterol.

[4] Eaden JA, Abrams KR, Mayberry JF (2001). The risk of colorectal cancer in ulcerative colitis: a meta-analysis. Gut.

[5] Lutgens MW, van Oijen MG, van der Heijden GJ, Vleggaar FP, Siersema PD, Oldenburg B (2013). Declining risk of colorectal cancer in inflammatory bowel disease: an updated meta-analysis of population based cohort studies. Inflamm Bowel Dis.

[6] Castaño-Milla C, Chaparro M, Gisbert JP (2014). Systematic review with meta-analysis: the declining risk of colorectal cancer in ulcerative colitis. Aliment Pharmacol Ther.

[7] Jess T, Rungoe C, Peyrin-Biroulet L (2012). Risk of colorectal cancer in patients with ulcerative colitis: a meta-analysis of population-based cohort studies. Clin Gastroenterol Hepatol.

[8] Watanabe T, Konishi T, Kishimoto J, Kotake K, Muto T, Sugihara K (2011). Ulcerative colitis-associated colorectal cancer shows a poorer survival than sporadic colorectal cancer: a nationwide Japanese study. Inflamm Bowel Dis.

[9] Higashi D, Futami K, Ishibashi Y (2011). Clinical course of colorectal cancer in patients with ulcerative colitis. Anticancer Res.

[10] Bargen JA (1928). Chronic ulcerative colitis associated with malignant disease. Arch Surg.

[11] Slaney G, Brooke BN (1959). Cancer in ulcerative colitis. Lancet.

[12] Bulow S (1980). Colorectal cancer in patients less than 40 years of age in Denmark 1943-1967. Dis Colon Rectum.

[13] Gyde SN, Prior P, Thompson H, Waterhouse JAH, Allan RN (1984). Survival of patients with colorectal cancer complicating ulcerative colitis. Gut.

[14] Klos C, Safar B, Wise PE, Hunt SR, Mutch MG, Birnbaum EH, Fleshman JW, Dharmarajan S. Impaired outcome colitis associated rectal cancer versus sporadic. J Surg Res 2016 (In Press). doi:10.1016/j.jss.2016.03.006. 10.1016/j.jss.2016.03.006 27451878

[15] van Heerden JA, Beart RW (1980). Carcinoma of the colon and rectum complicating chronic ulcerative colitis. Dis Colon Rectum.

[16] Lavery IC, Chiulli RA, Jagelman DG, Weakly FL (1982). Survival with carcinoma arising in mucosal ulcerativecolitis. Ann Surg.

[17] Aarnio M, Mustonen H, Mecklin JP, Jarvinen HJ (1998). Prognosis of colorectal cancer varies in different high-risk conditions. Ann Med.

[18] Jensen AB, Larsen M, Gislum M, Skriver MV, Jepsen P, Nørgaard B, Sørensen HT (2006). Survival after colorectal cancer in patients with ulcerative colitis: a nationwide population-based Danish study. Am J Gastroenterol.

[19] Shigaki K, Mitomi H, Fujimori T, Ichikawa K, Tomita S, Imura J, Fujii S, Itabashi M, Kameoka S, Sahara R, Takenoshita S (2013). Immunohistochemical analysis of chromogranin A and p53 expressions in ulcerative colitis-associated neoplasia: neuroendocrine differentiation as an early event in the colitis-neoplasia sequence. Hum Pathol.

[20] Yashiro M (2014). Ulcerative colitis-associated colorectal cancer. World J Gastroenterol.

[21] Kinugasa T, Akagi Y, Yoshida T, Ryu Y, Shiratuchi I, Ishibashi N, Shirouzu K (2010). Increased claudin-1 protein expression contributes to tumorigenesis in ulcerative colitis-associated colorectal cancer. Anticancer Res.

[22] Renz BW, Thasler WE, Preissler G, Heide T, Khalil PN, Mikhailov M, Jauch KW, Kreis ME, Rentsch M, Kleespies A (2013). Clinical outcome of IBD-associated versus sporadic colorectal cancer: a matched-pair analysis. J Gastrointest Surg.

[23] Meguid RA, Slidell MB, Wolfgang CL, Chang DC, Ahuja N (2008). Is there a difference in survival between right- versus left-sided colon cancers?. Ann Surg Oncol.

[24] Nitsche U, Zimmermann A, Spath C, Müller T, Maak M, Schuster T, Slotta-Huspenina J, Käse SA, Michalski CW, Janssen KP, Friess H, Rosenberg R, Bader FG (2013). Mucinous and signet-ring cell colorectal cancers differ from classical adenocarcinomas in tumor biology and prognosis. Ann Surg.

[25] Hyngstrom JR, Hu CY, Xing Y, You YN, Feig BW, Skibber JM, Rodriguez-Bigas MA, Cormier JC, Chang GJ (2012). Clinicopathology and outcomes for mucinous and signet ring colorectal adenocarcinoma:analysis from the National Cancer Data Base. Ann Surg Oncol.

[26] Kiran RP, Khoury W, Church JM, Lavery IC, Fazio VW, Remzi FH (2010). Colorectal cancer complicating inflammatory bowel disease: similarities and differences between Crohn's and ulcerative colitis based on three decades of experience. Ann Surg.

[27] Delaunoit T, Limburg PJ, Goldberg RM, Lymp JF, Loftus EV (2006). Colorectal cancer prognosis among patients with inflammatory bowel disease. Clin Gastroenterol Hepatol.

[28] Leowardi C, Schneider ML, Hinz U, Harnoss JM, Tarantino I, Lasitschka F, Ulrich A, Buchler MW, Kadmon M (2016). Prognosis of ulcerative colitis-associated colorectal carcinoma compared to sporadic colorectal carcinoma: a matched pair analysis. Ann Surg Oncol.

[29] Winther KV, Jess T, Langholz E, Munkholm P, Binder V (2003). Survival and cause-specific mortality in ulcerative colitis: follow-up of a population-based cohort in Copenhagen County. Gastroenterology.

[30] Oresland T, Bemelman WA, Sampietro GM, Spinelli A, Windsor A, Ferrante M, Marteau P, Zmora O, Kotze PG, Espin-Basany E, Tiret E, Sica G, Panis Y, Faerden AE, Biancone L, Angriman I, Serclova Z, de Buck van Overstraeten A, Gionchetti P, Stassen L, Warusavitarne J, Adamina M, Dignass A, Eliakim R, Magro F, D'Hoore A, European Crohn's and Colitis Organisation (ECCO) (2015). European evidence based consensus on surgery for ulcerative colitis. J Crohns Colitis.

[31] Uchino M, Ikeuchi H, Matsuoka H, Bando T, Hirata A, Yasukawa S, Takesue Y, Tomita N (2012). Surgical procedure for sporadic colorectal cancer in patients with mild ulcerative colitis. Case Rep Gastroenterol.

[32] Matsuoka H, Ikeuchi H, Uchino M, Bando T, Takesue Y, Nishigami T, Tomita N (2013). Clinicopathological features of ulcerative colitis-associated colorectal cancer pointing to efficiency of surveillance colonoscopy in a large retrospective Japanese cohort. Int J Colorectal Dis.

[33] Rogler G (2014). Chronic ulcerative colitis and colorectal cancer. Cancer Lett.

[34] Cairns SR, Scholefield JH, Steele RJ, Dunlop MG, Thomas HJ, Evans GD, Eaden JA, Rutter MD, Atkin WP, Saunders BP (2010). Guidelines for colorectal cancer screening and surveillance in moderate and high risk groups (update from 2002). Gut.

[35] Leighton JA, Shen B, Baron TH, Adler DG, Davila R, Egan JV, Faigel DO, Gan SI, Hirota WK, Lichtenstein D (2006). ASGE guideline: endoscopy in the diagnosis and treatment of inflammatory bowel disease. Gastrointest Endosc.

[36] Kornbluth A, Sachar DB (2010). Ulcerative colitis practice guidelines in adults: American College Of Gastroenterology, Practice Parameters Committee. Am J Gastroenterol.

[37] Eaden JA, Mayberry JF (2002). Guidelines for screening and surveillance of asymptomatic colorectal cancer in patients with inflammatory bowel disease. Gut.

[38] Farraye FA, Odze RD, Eaden J, Itzkowitz SH (2010). AGA technical review on the diagnosis and management of colorectal neoplasia in inflammatory bowel disease. Gastroenterology.

[39] Van Assche G, Dignass A, Bokemeyer B, Danese S, Gionchetti P, Moser G, Beaugerie L, Gomollón F, Häuser W, Herrlinger K (2013). Second European evidence-based consensus on the diagnosis and management of ulcerative colitis part 3: special situations. J Crohns Colitis.

